# Central Nervous System Involvement in Trichinellosis: A Systematic Review

**DOI:** 10.3390/diagnostics11060945

**Published:** 2021-05-25

**Authors:** Elena Cecilia Rosca, Raluca Tudor, Amalia Cornea, Mihaela Simu

**Affiliations:** 1 Victor Babes University of Medicine and Pharmacy Timisoara, Eftimie Murgu Sq. No. 2, 300041 Timisoara, Romania; tudor.raluca@yahoo.com (R.T.); amalia.cornea@yahoo.com (A.C.); mihaelasimu6713@gmail.com (M.S.); 2 Department of Neurology, Clinical Emergency County Hospital Timisoara, Bd. Iosif Bulbuca No. 10, 300736 Timisoara, Romania; 3 Neuroscience Research Center Timisoara, Clinical Emergency County Hospital Timisoara, Bd. Iosif Bulbuca No. 10, 300736 Timisoara, Romania

**Keywords:** *Trichinella*, central nervous system, encephalitis, meningitis, systematic review

## Abstract

We reviewed the evidence on features of central nervous system (CNS) involvement in trichinellosis, systematically searching five databases (to January 2021). We categorized clinical features based on their diagnostic value as warning signs for severe CNS infection (with outcome death) or non-specific signs (outcome improvement). They were suggestive of severe infection if they substantially raised death probability. The review included 87 papers published from 1906 through 2019, with data on 168 patients. Mydriasis, paraparesis, dysphagia, psychomotor seizures, or delirium present a 30–45% increased death likelihood. The best poor prognosis predictor is mydriasis (positive likelihood ratio 9.08). Slow/absent light reflex, diminished/absent knee reflexes, globally decreased tendon reflexes present a moderate increase (20–25%) of death risk. Anisocoria, acalculia, or seizures could also indicate an increased death risk. We provided a detailed presentation of clinical and paraclinical signs that alert physicians of a possible neurotrichinellosis, emphasizing signs that might indicate a poor prognosis.

## 1. Introduction

Trichinellosis is a parasitic disease caused by the consumption of raw meat infected with larvae of nematode in the genus *Trichinella*. The first report about the new findings of *Trichinella* was presented to the zoological society in London in 1835, when Sir Richard Owen, and his student, Sir James Paget, discovered the parasite during an autopsy [1]. Friedrich von Zenker reported the first acute case of human trichinellosis in 1860 [2]. He presented the dissemination mode of the parasites in the host by implicating the consumption of raw, infected pork as the vehicle of transmission. In 1906, Frothingham reported evidence of the central nervous system (CNS) involvement [3]. Later observations revealed the presence of *Trichinella* in the cerebrospinal fluid (CSF).

Human trichinellosis was reported in 55 (27.8%) countries around the world. *Trichinella* infection was documented in domestic animals, mainly in pigs, in 43 countries, and the wildlife of 66 countries [4]. A systematic review, including data between 1986 and 2009, reported an estimated global incidence rate of 469.2 to 985.3 cases per billion persons per year. The global mortality rate was 0.300–0.828 per billion persons per year [5].

The global distribution of *Trichinella* infection mirrors the geographical distribution of parasites in domestic animals (i.e., pig, horse, dog) and wild animals (i.e., boars, bear, badger, cougar, jackal, walrus, lizard and turtle), the dietary habits (i.e., eating raw meat), and the social and economic development of the countries [6,7]. After 1990, the socioeconomic changes in some Eastern European countries and Argentina, determined the reemergence of *Trichinella* infections in these areas. Nonetheless, in the last years, there was a significant decrease of the number of cases reported in the European Union countries, the United States, Canada and China [8]. Despite a general reduction of the number of cases with *Trichinella* infection, which is mainly due to the development of systematic regulations regarding the domestic animals (pigs), there has been an increase of the number of Trichinellosis cases due to the consumption of wild animals [7]. In the last decades, important *Trichinella* outbreaks were reported in South East Asia countries (e.g., Cambodia, Thailand, Vietnam) and South America (e.g., Argentina) [6]. In Africa, there is a low prevalence of human *Trichinellosis*, possibly due to the dietary and religious habits [4]. However, despite the religious laws forbidding the consumption of pork, *Trichinellosis* was also documented in Muslim countries such as Turkey [4]. 

After ingestion, under the influence of gastric secretions, the *Trichinella* larvae are released in the stomach and develop in the adult stage inside the enterocytes of small intestine. The newborn larvae are released into circulation, spreading through the tissues and organs. *Trichinella* spp. has an intracellular localization only in two different tissues, specifically, in enterocytes and skeletal muscle cells. It has a unique ability to transform the infected muscle cell and create a new type of cell in the host body, the so-called nurse cell [9]. From this place, the parasite induces the formation of muscle larvae excretory-secretory products (ES L1). The invasion of the host generates a complex immune response, which is better characterized by humoral rather than cellular responses (hence the importance of humoral response for diagnostic purposes) [10]. During the intestinal phase, the immune response includes both Th1 and Th2 responses. Initially, it induces a Th1 responses, followed by a dominant Th2 type of response. This later type of immune response is characterized by the production of high levels of cytokines IL-4, IL-5, IL-9, IL-10, IL-13, immunoglobulin E (IgE), and the mobilization of eosinophils, basophils, and mast cells [11]. The existence of Treg cells further characterizes the muscle phase. The chronic stimulation through ES L1 released during the muscle phase of *Trichinella* infection activates regulatory network elements. Immune events induced by Th2 and Treg cell types modulate the immune response of the host [11].

The *Trichinella* infection is characterized by peripheral blood and tissue eosinophilia and an increased total IgE levels, both being a consequence of Th2 activation. Eosinophilopoiesis begins in the bone marrow, followed by the migration of eosinophils through the circulatory system, infiltration of tissues with eosinophils at the inflammatory foci and, finally, degranulation, and cell death [12]. However, the protective role of eosinophiles against *Trichinella* remains under debate. Furthermore, when the number of eosinophils is increased, they can be toxic to host tissues. 

The *Trichinella* antigens stimulate dendritic cells and macrophages to interact with T cells. Activated T cells produce cytokines, including granulocyte-macrophage colony-stimulating factor (GM-CSF), IL-3 and IL-5. They will determine the proliferation and differentiation of precursors into eosinophils. The migration of the eosinophils from the bone marrow to the vessels is controlled mainly by IL-5. The *Trichinella* antigens might attract and affect eosinophils directly to interact with T cells. In addition, the alternatively activated macrophages (AAMs) also attract eosinophils through chitinase-like molecule (Ym1) and arginase 1 (Arg1). In addition, eosinophils are attracted by Galectin 9 (Gal-9), eotaxin-1, and eotaxin-2. The eosinophils around *Trichinella* in the tissues produce cytokines, cytotoxic secretory products, growth factors, lipid mediators, and neuro-mediators. These molecules were reported to play a role in larval killing, tissue injury, or tissue repair, together with various cells. The involvement of eosinophils in worm expulsion from the gut is still uncertain. Nonetheless, the increase of activated eosinophils is responsible for damage to the vascular walls probably because of the release of the major basic protein, which is elevated in patients with eosinophilia. Additionally, it explains tissue damage in the CNS and other tissues [12].

The clinical picture of *Trichinella* infection is heterogeneous, varying from asymptomatic to fatal, depending on the number and sites of larvae. The symptoms parallel the parasite cycle and the responses of the host. The first infection stage consists of non-specific gastroenteritis, occurring two to seven days after ingestion of infected food. The larva migration and invasion determine the characteristic clinical signs and symptoms of *Trichinella* infection. Larvae and their products cause a diffuse inflammatory reaction, with fever, headache, rash, focal edema, local inflammation, leukocytosis, and eosinophilia. The most affected muscles are the extraocular muscles, masseters, diaphragm, intercostals, deltoids, tongue, larynx, and neck muscles. In severe cases, diffuse and severe muscle involvement manifests with myalgia, especially of the diaphragm, calves, and forearms. Additionally, a characteristic finding is increased muscle consistency, with weakness [1]. 

The most severe complications are related to the CNS, heart, and lungs [13]. The CNS involvement was reported in 10–20% of patients and is usually associated with severe *Trichinella* infection. The reports on mortality vary between 8% [14] to 46% [15]. However, with the treatment with corticosteroids and benzimidazoles (e.g., mebendazole and albendazole), the number of fatal outcomes has decreased.

The clinical manifestations of CNS trichinellosis comprise diffuse encephalopathy and focal neurologic deficits. Cerebral invasion occurs during the second week, the larval migratory stage. The patients present with varying degrees of meningoencephalitis manifested by non-focal clinical signs. Focal CNS lesions occur during the third week, the encystment stage. The clinical signs often overlap with previous meningo-encephalitic symptoms. Only rarely are they the sole manifestation and include motor deficits (e.g., hemiparesis), cranial nerve deficits, cerebellar signs, aphasia, and seizures [13]. Additionally, patients may present occlusion of cerebral venous sinus, causing venous infarction or intracerebral hemorrhage [16]. Patients with focal brain damage may present significant diagnostic challenges, mainly if they are first seen with a focal cerebral picture.

The meningeal form frequently causes minimal mental and focal signs, while in the parenchymal form, patients present marked mental and focal signs with minimal meningeal involvement [13].

The Centers for Disease Control (CDC) case definition for trichinellosis comprises: (1) *Trichinella*-positive muscle biopsy or positive serologic test for trichinellosis in a patient with a clinical syndrome compatible with trichinellosis (including eosinophilia, fever, myalgia, and periorbital edema) or (2) in an outbreak, at least one person must meet criteria 1, with associated cases defined by a positive serologic test for trichinellosis or clinical symptoms compatible with trichinellosis (including eosinophilia, fever, myalgia, and periorbital edema) in individuals who shared the epidemiologically implicated meal or have consumed the implicated meat product [17].

The case definition for trichinellosis at the European Center for Disease Control states, “at least three of the following six: fever, muscle soreness and pain, gastrointestinal symptoms, facial edema, eosinophilia, and subconjunctival, subungual, and retinal hemorrhages” [10,17].

The differential diagnostic difficulties rarely arise in cases of “typical” acute trichinellosis. However, the cases with CNS involvement may represent a diagnostic challenge, especially in the lack of eosinophilia. In addition, the presentations of CNS infection are myriad, and the diagnosis can be elusive [18]. 

The drugs of choice in *Trichinellosis* are anthelmintics (albendazole or mebendazole). They should be given before the initiation of corticosteroids to prevent the effect of delayed expulsion of adult worms from the intestine. Their efficacity depends on the time delay between infection and the beginning of treatment, and it is likely dose-dependent [19]. Although no valid controlled studies have been performed, corticosteroids are frequently used to treat allergic manifestations, which occur at the beginning of the parenteral phase [10]. The corticotherapy is strongly recommended to suppress the vascular and muscle damage induced by eosinophil degranulation products [20]. In severe cases, corticosteroids were reported to reduce the course of the illness [21]; after their implementation, the number of fatal cases with neurological involvement has decreased significantly [19]. In addition, the neurological complications occurring in the early period of the illness were reported to be successfully treated with corticosteroids. Still, when these complications appear after one month, they can cause permanent sequelae [19].

In addition, practical recommendations have been published for severe and moderately severe diseases [10].

In the latest years, experts have developed risk-based approaches to control the presence of parasites in meat, requiring the re-evaluation of traditional practices and the assessment of regulatory and industry resources. In 2020, the Food and Agriculture Organization of the United Nations (FAO) and World Health Organization (WHO) published a report that provides spreadsheet models to get quantitative information needed by public health officials when evaluating different post-mortem hygiene programs for *Trichinella* spp. in meat [22]. These models enable the development of risk scenarios to assess the effect of changes to digestion testing and meat inspection on the risk of human Trichinellosis [22]. Applying the latest risk-based approach recommendations requires a re-evaluation of traditional practices and assessing regulatory and industry resources proportionate to risks. In addition, the link between control measures pre-and post-harvest along the food chain and public health outcomes will help risk managers locate the source of the infection at the farm, abattoir, processor, and consumer level for food safety interventions, according to the publication [22]. In addition, there are international recommendations for quality assurance in digestion testing programs for *Trichinella* [23]. Additionally, many countries have specific regulations for the inspection and control of the parasite, and there are different treatment methods to inactivate *Trichinella* larvae in meat, including cooking, irradiation, and freezing for some genotypes.

The present research aimed to systematically review and summarize the existing evidence on the CNS complications of *Trichinellosis* infection, described in case reports and case series. Although a case report provides only a descriptive result, systematic reviews of multiple cases allow narrative or quantitative synthesis, pattern recognition, and identification of unrecognized or rare associations. Furthermore, it can generate hypotheses for subsequent studies and advance medical knowledge.

## 2. Materials and Methods

The present systematic review was performed following the guidelines of the Preferred Reporting Items for Systematic Reviews and Meta-Analysis (PRISMA) [24] and the recent recommendations on the synthesis of case series and case reports [25]. 

We performed a computerized bibliographic search from inception to January 2021 on the following databases: MEDLINE/PubMed, EMBASE, Scopus, Web of Sciences, and Latin American and Caribbean Health Sciences Literature (LILACS). In addition, we performed a complementary manual search by checking reference lists of all relevant research papers to identify possible additional studies. The following keywords were used: “trichinosis” AND “brain disease” [MeSH], “trichinosis” AND “central nervous system disease” [MeSH], “*trichinella spiralis*” AND “brain disease” [MeSH], “*trichinella spiralis*” AND “central nervous system disease” [MeSH], and “neurotrichinosis”. These search terms were for PubMed. Searches in other data sources used similar versions of these terms, appropriate for each database. We did not utilize search filters (collection of terms to reduce the number needed to be screened) because we aimed to generate a comprehensive list of papers suitable for answering the research question. Additionally, we did not apply any language restrictions to our search. 

Two authors reviewed the title, abstract, and full text (when needed) of all retrieved research papers and assessed whether the study met the inclusion criteria. Disagreements were solved through discussion.

To perform a systematic review of the CNS involvement in trichinellosis, we selected all the studies reporting children or adults diagnosed with trichinellosis and meningeal or brain damage signs. The main types of eligible studies were case reports and case series reports. 

We excluded studies with no data on at least one aspect of CNS involvement, including clinical signs, CSF study, autopsy or biopsy, or neuroimaging. Additionally, we excluded studies in languages other than English and Spanish. 

Data were extracted to a proforma template that two authors initially piloted on a set of five randomly selected case reports and adjusted as necessary. Data were extracted by one reviewer and verified by a second reviewer.

Descriptive statistics of patient clinical and paraclinical variables were estimated. Case report data were grouped by the outcome (improvement or death). 

In addition, we reconstructed two-by-two tables based on clinical information in the case reports where an outcome was available. We calculated the likelihood ratios for a positive result (LR+), representing the probability that a patient with a specific clinical sign could die, as well as the likelihood ratios for negative results (LR−), referring to the probability that an individual presenting a specific clinical sign could present a less severe CNS infection, with improvement. For statistical calculations, we used the RevMan program.

The clinical features were categorized based on their diagnostic value as either warning signs for severe CNS infection (with outcome death) or non-specific signs for severe infection (with outcome improvement). Clinical features were considered suggestive for severe infection if, when positive, they substantially raised the probability of death (i.e., LR+ of more than 5.0).

The LR+ may range from 0 to infinity. The larger LR+, the more informative is the test. Findings with LR+ greater than 1 indicate an increased odd of having a particular condition in a patient with a positive result. The bigger the number, the more convincingly the finding suggests the presence of disease. If LR+ lies between 0 and 1, it argues against the diagnostic value of the test [26]. When diagnostic test accuracy results are around 1, it lacks diagnostic value [26]. A simple estimative method, independent of pretest probabilities and avoiding complex calculations, enables the clinician to make a bedside estimation: an LR+ of 2 causes a small change in disease likelihood, increasing the probability of the disease by 15%, one of 5 increases it 30%, determining a moderate change, and one of 10 increases it 45%, determining a large change in disease likelihood [26]. At the same time, the LR− indicates the odds of having a disease in patients with negative test results. If the LR− is smaller than 1, then the post-test probability of the disease decreases. The smaller the LR− the more informative is the test [26].

## 3. Results

**Figure 1:**
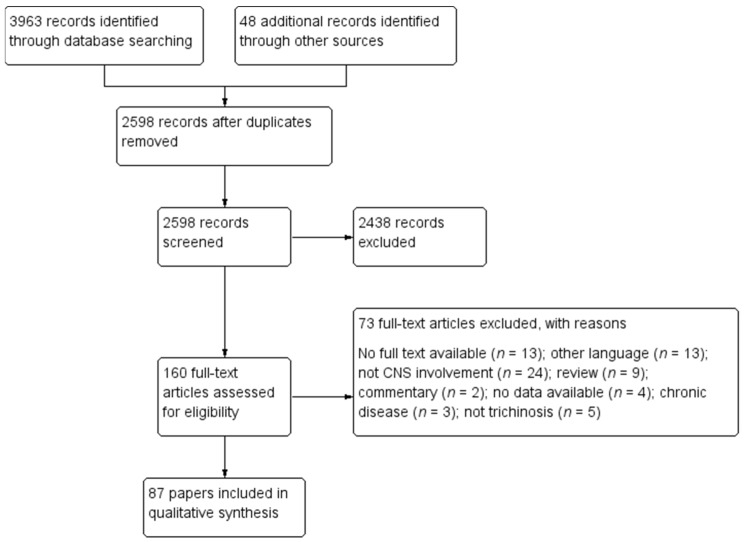
Study selection flowchart.

Our search resulted in 4011 records. From a total of 160 unique studies identified using the search strategy and assessed in full-text, we included in the present review 87 papers [3,13,14,15,16,18,20,27,28,29,30,31,32,33,34,35,36,37,38,39,40,41,42,43,44,45,46,47,48,49,50,51,52,53,54,55,56,57,58,59,60,61,62,63,64,65,66,67,68,69,70,71,72,73,74,75,76,77,78,79,80,81,82,83,84,85,86,87,88,89,90,91,92,93,94,95,96,97,98,99,100,101,102,103,104,105,106]. The PRISMA diagram describing the selection process of studies is detailed in Figure 1.

Seventy-three studies were excluded for the following reasons: no full text was available (*n* = 13); the paper was in other languages (*n* = 13); the patients did not present CNS involvement (*n* = 24); the article was a review (*n* = 9) or a commentary (*n* = 2); no data was available (*n* = 4); the patients presented chronic disease (*n* = 3), and the disease was not trichinellosis (*n* = 5).

The characteristics of the included case reports are summarized in Supplementary Table S1.

The present review included 168 cases of patients with meningeal or brain disease due to *Trichinella* infection. Acute cerebral sinus thrombosis was reported in five papers [16,41,83,92,104]. The rest were reports of meningitis or neurotrichinellosis. The year of publication ranged from 1906 through 2019.

### 3.1. Clinical Findings

The clinical picture was presented in 162 cases (Table 1, Supplementary Table S1).

The most frequent clinical symptoms were headache (24.69% of patients), confusion (14.2%), disorientation (11,73%), delirium (9.87%), and meningeal signs including positive Kerning sign (14.8%) and neck stiffness (13.58%). Central motor deficits were also frequent (19.14% hemiparesis, 10.49% tetraparesis, 8.02% monoparesis, and 1.23% paraparesis). They are associated with pathological tendon reflexes (brisk or, on the contrary, diminished) and positive Babinski sign (unilateral in 8.02% of cases, bilateral in 9.88% of cases, doubtful in 0.62% of patients). Sensitive impairment was rare, with hemihypoesthesia in 3.70% of patients and subjective symptoms (e.g., numbness, dysesthesia) in 3.70% of cases. Cerebellar impairments were reported in 9.25% of patients. Cranial nerves were also affected, with third nerve palsy (4.94%), sixth nerve palsy (5.56%), peripheral facial palsy (0.62%), dysphagia (1.23%), and dysarthria (0.62%). Pupillary abnormalities consisted of mydriasis (3.09%), anisocoria (3.09%), and absent or slow light reaction (4.94%).

Hemiballismus, an involuntary movement disorder, was reported in one case (0.62%). Epileptic seizures were present in some rare instances (generalized seizures 2.46%, jacksonian seizures 1.85%, petit mal 1.23%, and psychomotor seizures 1.85%). 

Cognitive problems included memory impairments, affecting mainly the recent memory (10.49%), aphasia (6.79%), acalculia (1.85%), apraxia (0.62%), and anosognosia (0.62%). Behavioral disturbances consisted of apathy (4.94%), anxiety (1.23%), insomnia (2.47%), agitation (3.08%), and irritability (2.47%). Psychiatric disturbances (e.g., psychosis, bizarre mental attitude, hallucinations, paranoid reactions) were present in 12.96% of patients. 

Of the 162 cases, coma was reported in 6.79%, stupor in 2.47%, lethargy in 6.17%, somnolence in 6.17%, and drowsiness in 6.17%.

A detailed presentation of all the reported symptoms is presented in Table 1.

### 3.2. Clinical Findings in Patients with Reported Outcome

The outcome was published in 124 cases: 20 patients died, and 104 improved. 

Among 20 patients who died, the clinical picture was presented in 17. The most frequent symptoms consisted of headache (41.17%), disorientation (41.17%), followed by motor deficits (hemiparesis 23.53%, tetraparesis 17.65%, and paraparesis 5.88%), diminished or absent tendon reflexes (globally 23.53%, or knee reflexes 11.76%), Kerning sign (17.56%), pupillary abnormalities (mydriasis 17.6%, anisocoria 11.76%, and slow or absent light reflex 17.65%), aphasia (17.65%), and drowsiness (17.65%). A complete list with the symptoms reported in patients who died is presented in Table 2. 

### 3.3. Cerebrospinal Fluid Analysis

The CSF findings were reported in 107 patients (see Table 3).

Almost half of the patients presented normal findings (41.12%). The pressure was reported increased by 10.28% of cases and decreased in a few cases (2.80%). The cells found in the CSF were lymphocytes (14.95%) or erythrocytes (3.74%). Proteins were increased in 14.95% of cases and decreased in 5.60% of patients. Glucose level was rarely modified (increased—4.67%, and decreased—0.93%). Authors found a CSF positive for *Trichinella* sp. larvae in 18.69% of patients. Anti-*Trichinella* antibodies were detected in the CFS of one patient.

Among the 20 patients who died, the CSF analysis was reported in 14. Four reports comprised only of data on the presence of *Trichinella* sp. larvae, without any data on the other CSF parameters. Among the remaining ten patients, four presented completely normal CSF findings. One patient presented increased pressure, one had decreased CSF pressure, and eight presented normal pressure. Lymphocytes were found in two cases; the other two patients were reported to have CSF cells, but their type was not specified. Two patients presented no cells in the CSF. The protein level was increased in three cases and normal in seven cases. Eight patients presented normal glucose levels, one an increased level, and one a decreased level. In total, the presence of *Trichinella* sp. larvae was assessed in six cases, with three positive and three negative findings. 

### 3.4. Blood Eosinophils

The blood level of eosinophils was reported in 107 patients. In the group with clinical improvement, consisting of 96 patients, the majority (89.58%) presented eosinophilia. In the group of the patients who died, data on eosinophils count was provided in 11 patients. Eosinophilia was present in 45.45% of cases, the rest of 54.55% being without increased eosinophils in the blood.

### 3.5. Autopsy and Biopsy Findings

Autopsy data was published in 14 patients, the last report being in 1993 [20]. The findings consisted of diffuse inflammatory changes, typical for acute encephalitis [36], areas of cellular infiltration comprised of endothelial cells, glial cells, and lymphocytes [3,36,42]. Some authors also reported mild brain hemorrhages without marked cellular reaction [3,80], but others noted the absence of hemorrhages [36]. In addition, autopsy findings included nodules distributed through the hematogenous elements, harboring *Trichinella* [36,46]. Other authors also reported the presence of *Trichinella* larvae outside the vessels [3,30,41,42,71]. Nonetheless, other authors report the absence of *Trichinella* in the brain [16,20,48]. The nodules were found mainly in the subcortical white matter [46]. Etat criblé, with numerous round and oval cavities, was present in some patients (Supplemental material S1) [20,32,46].

Other autopsy findings consisted of congestion and hyperemia of the brain vessels [36,42,43,46], perivascular infiltrates [46], and meningeal congestion [32,36,42,43].

One paper, published in 2007, reported data on brain biopsy [98]. The authors described the presence of active vasculitic changes, with focal necrosis of the vessels and deposits of eosinophils [98].

### 3.6. Neuroimaging Findings

Neuroimaging was performed in 49 patients. Eight patients had a skull radiograph. One revealed a brain calcification [38], and the rest were normal. CT scans were performed in 27 cases. Five patients presented a normal CT scan. In 22 cases, the CT revealed some abnormalities, consisting mainly of hypodense lesions, situated with predilection in the white matter. Some authors reported lesions with nodular enhancement [89] or non-specific enhancement [83,87]. One author found cerebral hemorrhage in a patient who presented on autopsy cerebral sinus thrombosis [16]. Calcification rings were described in 1 patient [89]. Sinus thrombosis was present in one patient [104]. 

A brain MRI was reported in 14 patients. Most authors reported the presence of high signal lesions in the T2 weighted sequences, located in the white matter [20,87,94,97,98,101,102,105,106]. In T1 sequences, patients presented a slight signal increase [97] or hypointense lesions [101]. The DWI sequences revealed a restricted diffusion, suggesting ischemia [97,101]. In apparent diffusion coefficient (ADC) sequences, the lesions were hypointense [98]. Interestingly, some papers reported that the lesions were situated in the border zones of distal fields of major cerebral arteries [97,98,105,106]. Although most of the lesions were reported to be in the white brain matter, the MRI scans also detected lesions at the white matter and grey matter interface, in the cortex [102], cerebellum [98,101,105,106], and thalamus [106]. Furthermore, McDonald et al. [101] observed that the lesions were distributed mainly in the frontal and parietal lobes, with fewer lesions in the temporal and occipital lobes and cerebellum. 

Some of the lesions found on MRI presented rim enhancement [101], or peripheral enhancement, indicating a breakdown of the blood-brain barrier [98], focal nodular enhancement [94], or focal gyriform enhancement [94]. 

In one case of cerebral sinus thrombosis, the patient presented on MRI a hemorrhagic infarct [92].

Among the patients with neuroimaging data, only three presented a severe outcome (death): one had cortical sinus and venous thrombosis, with a secondary hemorrhage [16], one presented on the CT scan multiple unenhanced hypointensities located bilaterally in the white matter [20], and one had a hemorrhagic infarct secondary to the superior sagittal and lateral sinus thrombosis [92]. Nonetheless, due to the low number of neuroimaging data in severe cases, it was impossible to ascertain if any neuroimaging features could indicate a severe prognosis.

### 3.7. Statistical Analysis

The probability of a severe CNS infection (with outcome death), based on LR+ of clinical neurological features, was calculated based on data from 120 patients. Among them, 17 died, and 103 improved. The highest LR+ was obtained for mydriasis (9.08), paraparesis (6.05), dysphagia (6.05), psychomotor seizures (6.05), and delirium (5.30). A moderate increase of risk of death was indicated by the presence of clinical signs with LR+ between 3 and 5: acalculia (3.02), diminished or absent knee reflex (3.02), globally decreased tendon reflexes (3.46), anisocoria (4.03), slow or absent light reflex (4.03), and seizures (4.03). The lowest LR+ was found for confusion (0.27), monoparesis (0.50), unilateral Babinski sign (0.55), neck stiffness (0.60), recent memory impairments (0.60), lethargy (0.75), sixth cranial nerve palsy (0.75), depression (0.86), and apathy (0.86). The detailed presentation of the LR is presented in Table 4.

## 4. Discussion

The present systematic review enabled us to make several key observations. 

The most frequent clinical features of the CNS involvement in *Trichinella* infection consist of some non-specific meningo-encephalitic findings, such as headaches, confusion, spatial and temporal disorientation, and meningeal signs, including neck stiffness and Kerning signs. Additionally, patients may present focal brain damage, indicated mainly by motor deficits, most frequently hemiparesis. Tendon reflexes might be brisk or diminished, and the Babinski sign is also present in almost one-quarter of the patients. Cerebellar and cranial nerve involvement is relatively rare, and so are seizures. 

Psychiatric and behavioral disturbances are not very frequent. Cognitive impairments consist mainly of deficits in recent memory [20,46,61,62,76,79,103], resembling Korsakoff syndrome and aphasia [20,34,41,70,76,94,99,101,105].

Interestingly, in patients who died, the headache, neck stiffness, and positive Kerning sign rates were quite similar to those with a favorable outcome. However, delirium was present in almost half of the deceased cases (41.17%), compared to 9.87% in the group of all patients. Additionally, the tendon reflexes were diminished in almost half of the patients with unfavorable outcome. Among 24 patients with decreased knee tendon reflexes, 11 died. Mydriasis and absent or slow light reflex were reported in a higher percentage of patients who died than patients with improvement. (17.65% mydriasis and 17.5% slow light reflex vs. 3.09%, respectively 4.94%). Among 13 patients with mydriasis or impaired light reflex, six of them died.

The clinical signs with an LR+ between 5 and 10, indicating an increased chance of death with 30–45%, included mydriasis, paraparesis, dysphagia, psychomotor seizures, and delirium. The best predictor of a poor prognosis was mydriasis (LR+ of 9.08). Although some clinical signs such as diminished knee reflexes [13,107] and absent or slow light reflex [107] were earlier postulated to indicate a poor prognosis, we found them to present a moderate increase in death risk of 20–25%: diminished or absent knee reflex (3.02), globally decreased tendon reflexes (3.46), slow or absent light reflex (4.03). Additionally, we found that anisocoria, acalculia, or seizures could also indicate an increased risk of death.

Interestingly, clinical findings that usually for a neurologist are red flags, such as bilateral Babinski sign, lethargy, stupor, or positive Kerning sign, present a low LR+. 

The CSF findings were normal in 44 (41.12%) patients. The rest of the samples presented mainly increased pressure (10.28%) and increased proteins (14.95%). In most cases that presented cells in the CSF, they were leucocytes (14.95%). Glucose levels were abnormal in 5.6% of cases. *Trichinella* larvae were found in less than one-quarter of the patients (18.69%). In patients who died, CSF was normal in 40%, the pressure and glucose levels were normal in 80%, cells were in a normal range in 60%, and proteins were found normal in 70% of cases. Therefore, the absence of pathological findings in CSF does not exclude a poor prognosis.

Blood eosinophilia was present in 89.58% of all cases. Nonetheless, approximately half of the patients who died (45.45%) did not present an increase in eosinophils count. Therefore, it may be postulated that a lack of eosinophilia indicates a poor outcome in patients with a severe symptomatology [100], but further investigation of this paraclinical parameter is necessary. 

The CT findings in neurotrichinellosis are non-specific, with multiple hypodensities in the white matter. Nonetheless, a normal CT scan does not exclude CNS involvement. The imaging method of choice should be MRI, which is more sensitive in assessing the extent of brain damage. Border-zone brain lesions may be indicative of neurotrichinellosis. In particular, a patient with eosinophilia and border-zone ischemic lesions should be investigated for idiopathic hypereosinophilic syndrome or brain infection (trichinellosis, filariasis, or schistosomiasis) [97,105,108]. As indicated by autopsy reports, the majority of lesions are situated in the white matter. Notably, lesions of the thalamus, cerebellum, and brain stem were less frequent. In addition, rim or nodular enhancement suggests a possible neurotrichinellosis. 

Our systematic review included a comprehensive literature search, including articles published in the last 115 years. Nevertheless, our findings are limited by the quality and breadth of the data in the case reports, which was not uniform or consistent in all papers. In addition, after 1969, the antihelmintic treatment introduction could have hypothetically modified the clinical picture of cerebral trichinellosis. Nonetheless, the clinical signs reported in neurotrichinellosis after this date were relatively similar to previous cases. Although the number of deaths decreased, it is noteworthy that the clinical signs that we found as indicating a poor prognosis were reported more rarely.

Most importantly, the analysis of case series and reports can suggest hypotheses. Therefore, clinicians should be aware of the large number of cases reported in the literature and the multitude of CNS manifestations. The evidence provided should alert physicians of the possible CNS involvement in trichinellosis infection and some clinical signs that might indicate a poor prognosis. However, further studies are necessary for the diagnostic value of clinical and paraclinical features of neurotrichinellosis to identify severe CNS infection. 

Despite the limitations mentioned above, our study represents the first systematic review of the literature published in this field and outlines an accurate state of knowledge of cerebral and meningeal implications in acute *Trichinellosis*.

## Figures and Tables

**Table 1:** Clinical symptoms and signs of included cases.

Clinical Sign/Symptom		Number of Cases (% of Total; *N* = 162)	Deaths
Headache		40 (24.69%)	7
Neck stiffness		22 (13.58%)	2
Kerning sign		24 (14.8%)	16
Meningeal cry		2 (1.23%)	0
Confusion		23 (14.2%)	1
Delirium		16 (9.87%)	7
Disorientation		19 (11.73)	3
Agitation		5 (3.08%)	0
Insomnia		4 (2.47%)	0
Irritability		4 (2.47%)	1
Psychiatric symptoms		21 (12.96%)	0
Mutism		1 (0.62%)	0
Euphoria		2 (1.23%)	0
Depression		8 (4.94%)	1
Anxiety		2 (1.23%)	0
Apathy		8 (4.94%)	1
Bradypsychia		4 (2.47%)	0
Memory impairment (recent memory)		17 (10.49%)	1
Impaired abstract thinking		4 (2.47%)	0
Impaired concentration		1 (0.62%)	0
Anosognosia		1 (0.62%)	0
Apraxia		1 (0.62%)	0
Acalculia		3 (1.85%)	1
Aphasia		11 (6.79%)	3
Tetraparesis		17 (10.49%)	3
Hemiparesis		31 (19.14%)	5
Paraparesis		2 (1.23%)	1
Monoparesis		13 (8.02%)	1
Brisk tendon reflexes	Globally	9 (5.56%)	1
	On the hemiparesis side	4 (2.47%)	0
	Knee	3 (1.85%)	0
	Lower limbs	7 (9.87%)	0
Ankle clonus		9 (5.56%)	0
Rotulian clonus		1 (0.62%)	0
Diminished/absent tendon reflexes	Globally	11 (6.79%)	4
	Lower limbs	5 (3.09%)	5
	Knee	8 (4.94%)	2
	Ankle	3 (1.85%)	0
	Unilateral	1 (0.62%)	0
Babinski sign	Unilateral	13 (8.02%)	1
	Bilateral	16 (9.88%)	3
	Doubtful	1 (0.62%)	0
Sensitive impairment	Hemihypoesthesia	6 (3.70%)	0
	Subjective symptoms	6 (3.70%)	0
Cerebellar signs		15 (9.25%)	0
Cranial nerves	Third nerve palsy	8 (4.94%)	1
	Sixth nerve palsy	9 (5.56%)	1
	Peripheral facial palsy	1 (0.62%)	0
	Dysphagia	2 (1.23%)	0
	Pseudobulbar dysphagia	2 (1.23%)	1
	Dysarthria	1 (0.62%)	0
	Unspecified	2 (1.23%)	1
Mydriasis		5 (3.09%)	3
Anisocoria		5 (3.09%)	2
Absent/slow light reaction		8 (4.94%)	3
Ocular bobbing		1 (0.62%)	0
Horner syndrome		1 (0.62%)	0
Visual impairment		10 (6.17%)	0
Hemiballismus		1 (0.62%)	0
Seizures	Generalized	4 (2.46%)	1
	Jacksonian type	3 (1.85%)	1
	Petit mal	2 (1.23%)	0
	Psychomotor	3 (1.85%)	1
	Unspecified	2 (1.23%)	1
Coma		11 (6.79%)	3
Stupor		4 (2.47%)	1
Somnolence		10 (6.17%)	0
Lethargy		10 (6.17%)	1
Obnubilation		1 (0.62%)	0
Drowsiness		10 (6.17%)	3
Dizziness		5 (3.09%)	0
Frontal signs (unspecified)		3 (1.85%)	0
Encephalitic signs (unspecified)		2 (1.23%)	0

**Table 2:** Clinical signs and symptoms of cases with reported outcome.

Clinical Sign/Symptom		Number of Cases with Outcome Death (% of Total; *N* = 17)	Number of Cases with Outcome Improvement (*N* = 103)
Headache		7 (41.17%)	33
Neck stiffness		2 (11.76%)	20
Kerning sign		2 (17.65%)	8
Confusion		1 (5.88%)	22
Delirium		7 (41.17%)	8
Disorientation		3 (17.65%)	17
Depression		1 (5.88%)	7
Apathy		1 (5.88%)	7
Memory impairment (recent memory)		1 (5.88%)	10
Acalculia		1 (5.88%)	2
Aphasia		3 (17.65%)	8
Tetraparesis		3 (17.65%)	14
Hemiparesis		6 (35.29%)	22
Paraparesis		1 (5.88%)	1
Monoparesis		1 (5.88%)	12
Brisk tendon reflexes	Globally	1 (5.88%)	5
Diminished/absent tendon reflexes	Globally	4 (23.53%)	7
	Knee	2 (11.76%)	4
Present tendon reflexes		1 (5.88%)	1
Babinski sign	Unilateral	1 (5.88%)	11
	Bilateral	3 (17.65%)	14
Cranial nerves	Third nerve palsy	1 (5.88%)	5
	Sixth nerve palsy	1 (5.88%)	8
	Dysphagia	1 (5.88%)	1
Mydriasis		3 (17.65%)	2
Anisocoria		2 (11.76%)	3
Absent/slow light reaction		3 (17.65%)	5
Seizures	Generalized	1 (5.88%)	2
	Jacksonian type	1 (5.88%)	3
	Psychomotor	1 (5.88%)	1
	Unspecified	1 (5.88%)	0
Coma		3 (17.65%)	8
Stupor		1 (5.88%)	3
Lethargy		1 (5.88%)	8
Drowsiness		3 (17.65%)	7

**Table 3:** Cerebrospinal fluid (CSF) findings of included cases.

CSF Parameter		No. of Cases (% of Total; *N* = 107)
Pressure	increased	11 (10.28%)
	decreased	3 (2.80%)
Cells	Lymphocytes	16 (14.95)
	Erythrocytes	4 (3.74%)
	Unspecified	3 (2.80)
Proteins	Increased	16 (14.95%)
	Decreased	6 (5.60%)
Glucose	Increased	5 (4.67%)
	Decreased	1 (0.93%)
Positive for *Trichinella* sp. larvae		20 (18.69%)
Anti-trichinella antibodies		1 (0.93%)
Normal findings		44 (41.12%)

**Table 4:** Data on the probability of a severe CNS infection (with outcome death) based on likelihood ratios of clinical neurologic features.

Symptom		TP	FP	FN	TN	LR+	LR−
Headache		7	33	10	70	1.28	0.86
Neck stiffness		2	20	15	83	0.60	1.09
Kerning sign		2	8	15	95	1.51	0.95
Confusion		1	22	16	81	0.27	1.19
Delirium		7	8	10	95	5.30	0.63
Disorientation		3	17	14	86	1.06	0.98
Depression		1	7	16	96	0.86	1.00
Apathy		1	7	16	96	0.86	1.00
Memory impairment		1	10	16	93	0.60	1.04
Acalculia		1	2	16	101	3.02	0.95
Aphasia		3	8	14	95	2.27	0.89
Tetraparesis		3	14	14	89	1.29	0.95
Hemiparesis		6	22	11	81	1.65	0.82
Paraparesis		1	1	16	102	6.05	0.95
Monoparesis		1	12	16	91	0.50	1.06
Brisk tendon reflexes (globally)		1	5	16	98	1.21	0.98
Diminished tendon reflexes	Globally	4	7	13	96	3.46	0.82
	Knee	2	4	15	99	3.02	0.91
Babinski sign	Unilateral	1	11	16	92	0.55	1.05
	Bilateral	3	14	14	89	1.29	0.95
Third cranial nerve palsy		1	5	16	98	1.21	0.98
Sixth cranial nerve palsy		1	8	16	95	0.75	1.02
Dysphagia		1	1	16	102	6.05	0.95
Mydriasis		3	2	14	101	9.08	0.83
Anisocoria		2	3	15	100	4.03	0.90
Absent/slow light reflex		3	5	14	98	3.63	0.86
Jacksonian seizures		1	3	16	100	2.01	0.96
Psychomotor seizures		1	1	16	102	6.05	0.95
Any type of seizures		4	6	13	97	4.03	0.81
Coma		3	8	14	96	2.27	0.89
Stupor		1	3	16	100	2.01	0.96
Lethargy		1	8	16	95	0.75	1.02
Drowsiness		3	7	14	96	2.59	0.88

Abbreviations: TP—true positive; FP—false positive; FN—false negative; TN—true negative; LR+—positive likelihood ratio; LR−—negative likelihood ratio.

## Data Availability

All data are available as part of the article and Supplementary materials (Table S1).

## Supplementary Materials

The following is available online at www.mdpi.com/xxx/s1/. Table S1: Characteristics of the included case reports.
