# Supplementary material for: Central Nervous System Involvement in Trichinellosis: A Systematic Review
Source: Diagnostics (Basel). 2021 May 25;11(6):945. doi: 10.3390/diagnostics11060945 (PMC8227095; doi:10.3390/diagnostics11060945)
Supplement: Supplementary file 1 [file diagnostics-11-00945-s001.zip › diagnostics-1182558-supplementary.pdf]

Supplementary Table 1. Characteristics of included cases.

Study	Year	No of cases	Age	Clinical signs	CSF	Autopsy	Neuroimaging	Other
Frothingham	1906	1 F	-	-	N/A	Many areas of slight hemorrhage into the brain substance, around which there is no marked cellular reaction.<br>Areas of cellular infiltration. The cells are mainly endothelial and neuroglia cells with an occasional leucocyte or lymphocyte.<br>In such an area, part of a trichina embryo sometimes appears outside the vessels.<br>Around these areas, the neuroglia presents a slight reaction (phosphotungstic acid hematoxylin stain).	N/A	Outcome: death
Van Cott	1914	1 F	21	Headache<br>Mydriasis<br>Kerning sign<br>Diminished knee reflexes	Pressure: moderate<br>Albumin: normal<br>Sediment: slight greyish-white<br>Cells: Lymphocytes<br>Young actively moving trichinae +	N/A	N/A	Outcome: death
Lintz	1916	3	-	-	Clear<br>Albumin: faint trace<br>Globulin absent<br>Trichinae embryos: 1-4/field<br>Occasional lymphocytes	N/A	N/A	Blood: leukocytosis with eosinophilia
Elliot	1916	1 F	14	Headache, vomiting<br>Kerning sign	Pressure: moderately increased<br>Clear<br>Cells: 3 cells/mm^3<br>Actively motile trichina larvae +	N/A	N/A	Blood: Leukocytosis with eosinophilia
Salzer	1916	14	-	Kerning – 100%<br>Reflexes – abolished in lower limbs – 100%	Trichinae + (8 cases)<br>In one case trichinae were still found in the CSF of a child 3 years of age, 3 months after clinical recovery.	Trichinae occurred abundantly in the brain, and on injection of such tissue into animals the disease can be produced, the eosinophilia being more marked than any other form of production of the disease.	N/A	The leukocytosis diminished as the eosinophilia increased.
Cummins	1916	1		-	Cells: absent<br>Trichina embryo +	N/A	N/A	Blood: leukocytosis with eosinophilia<br>Outcome: improvement
Bloch	1917	1	23	Headache	Trichinella: absent	Intense meningeal congestion,	N/A	Outcome: death

				Delirium<br>Generalized and jacksonian seizures<br>Left hemiparesis<br>Bilateral pyramidal signs<br>Coma		edema of the posterior half of the right hemisphere and slight opacity of the pia.<br>No Trichinae in the brain, no infiltrations, nodules or syncitia,<br>Marked état criblé, glia reticulum and neuronophagic phenomena, with other signs of severe ganglion cell lesions, but without accumulation of lipoids.		
Meyer	1918	1F	6 years	Meningitis: Headache, vomiting, marked irritability, rigidity of the neck, Kerning sign<br>Absent knee reflexes	Pressure: increased<br>Clear<br>58 cells/mm^3<br>Lymphocytes present<br>Organisms -	N/A	N/A	Blood: leukocytosis with eosinophilia<br>Outcome: improvement
		1 M	8 years	Meningitis: headache, vomiting, Kerning sign<br>Brisk knee jerks<br>Babinski doubtful	Pressure: increased<br>Clear<br>50 cells/mm3<br>Lymphocytes +	N/A	N/A	Blood: leukocytosis with eosinophilia<br>Outcome: improvement<br>Associated: Herpes
		1 F	12 years	Meningitis: neck rigidity<br>Kerning sign +, Brudzinski sign – absent knee reflexes	Pressure: increased<br>Clear<br>240 cells/mm3<br>Lymphocytes +<br>Albumine -<br>Actively motile<br>ous trichinae +<br>Trichina larvae +.			Blood: leukocytosis with eosinophilia<br>Associated: Herpes
Sterling	1925	1 F	26 years	Headache<br>Hemiplegia right<br>Hemihypoesthesia<br>Motor aphasia<br>Brisk lower limb reflexes	N/A	N/A	N/A	Outcome: improved
		1 M	56	Unconsciousness, followed by left hemiparesis<br>Brisk lower limb reflexes	-	-	-	Outcome: improved
Horlick	1929	1 F	52	Headache<br>Stupor<br>Left spastic hemiparesis<br>Incoherent speech<br>Disorientation<br>Brisk knee jerks, biceps, triceps and achilles reflexes.<br>Bilateral Babinski sign	Trichinella +	N/A	N/A	Blood: leukocytosis with eosinophilia<br>Outcome: death
Hassin	1926	1 M	14	Headache<br>Delirium	The spinal fluid removed at the necropsy<br>Numerous trichina larvae	**Macroscopic:**<br>The pia of the brain was transparent, with marked congestion in some areas;<br>The sulci, especially in the frontal lobe, were gaping;<br>The gyri were of normal size and	N/A	Leukocytosis with eosinophilia<br>Outcome: death:

not flattened.
Normal base of the brain
The ventricles were not dilated, the cortical gray matter was sharply defined from the white substance.
Congested cerebral parenchyma, without foci of softening or hemorrhage.
Microscopic:
The pia-arachnoid, throughout the cortex and the base of the brain, was markedly infiltrated, some areas (occipital and Ammon's horn) more, some (frontal lobe, cerebellum) less.
The infiltrative elements in some regions (cerebellum), were mostly large lymphocytes in the form of homogeneous, pale, round or slightly oval bodies containing an eccentric nucleus fairly rich in chromatin. In other areas mesothelial cells predominated and often formed clusters. As a rule, these types of cells were mixed with a great variety of other elements—plasma cells, fat granule bodies (gitter cells), fibroblasts and macrophages, which sometimes contained within their vacuoles lymphocytes or polymorphonuclear leukocytes. The latter were mostly eosinophils
These occasionally formed dense masses or foci.
Typical embryos of Trichina were found. They were absent in the blood vessels, which were slightly infiltrated and markedly congested.
Hemorrhages and blood pigment granules were absent, but red cells, mostly well preserved, were abundantly scattered among the cellular elements
The pia-arachnoid changes may be designated as an acute non--suppurative leptomeningitis.
Some areas in the frontal lobe of the brain and in the pons exhibited numerous small, round or oblong

cavities. These were, for the most part empty; some contained a collapsed blood vessel, the walls of which were sometimes connected with the surrounding parenchyma by strands of glia tissue.
In rare instances, the cavities were adjacent to or surrounded ganglion cells, while the brain substance itself appeared slightly rarefied.
Disintegration was quite evident in the basal portions of the pons, where lacunae were present.
Small islands or nodules consisting of glia cells and enclosing portions of Trichinae embryos, while the adjacent nerve tissue was rarefied, showing as glia reticulum.
The foregoing cell conglomerations or nodules were also present in brain areas that showed no disintegration. They appeared as granulomas and were cellular.
The nodules invariably harbored a Trichina embryo, whole or a fragment of it, but no blood vessels or ganglion cells. They were scattered in large numbers throughout the white brain substance, basal ganglia, pons and cerebellum, but were absent in the cortex.
Rarely a stray Trichina was encountered in the parenchyma.
Pronounced changes in the blood vessels. Throughout the cortex, midbrain, medulla and other regions the arteries and the veins were greatly infiltrated.
The blood vessels were also densely hyperemic, and their endothelial as well as adventitial cells markedly hypertrophied and hyperplastic.
Hemorrhages, in the form of small foci, occasionally were present around a capillary or small blood vessel.
In contrast to the larger blood vessels, the capillaries exhibited no

						signs of adventitial infiltration, but their endothelial layer was markedly hypertrophied.<br>The capillaries and the arteries and veins uniformly showed the presence of lipoid substances<br>In the spaces outside the tufts or choroid villi, lying freely in the cavity of the fourth and lateral ventricles, were present Trichina larvae<br>Histologic changes may be defined as diffuse, degenerative and inflammatory, with marked hyperplastic phenomena in the pia-arachnoid, choroid plexus and glia (syncitia and nodule formation). Typical of acute encephalitis, they were associated with the presence of Trichina embryos in the cerebral parenchyma, nodules, infiltrated adventitial and subarachnoid spaces and in the cerebral ventricles. These changes strikingly resemble those of other types of acute nonsuppurative encephalitis.		
Salan	1928	1 F	16	Neck stiffness<br>The optic disks were congested, with blurred margins (right > left).<br>Weakness of the left external rectus muscle<br>Right central facial weakness<br>Right hemiparesis<br>Bilateral Babinski sign<br>Tendon reflexes right > left	Increased pressure<br>Absent cells<br>Increased glucose (mild)<br>Trichinella larvae +	N/A	N/A	Trichina + in blood, muscle<br>Blood: mild anemia, a slight leukocytosis and a marked eosinophilia<br>- 23 days after admission the trichina larvae disappeared from the CSF<br>Outcome: improvement
Dandy	1929	1		Epileptic seizures	N/A	N/A	The roentgenologic study revealed a calcific focus in the subrolandic area	-
Stoll	1929	1 M	21	Diplopia<br>Left ptosis<br>Bilateral sixth nerve palsy	Cells: 92 lymphocytes<br>Trichinae: absent	N/A	N/A	Blood: eosinophilia
		1 M	20	Diplopia when he looked sideways	Cells: 90 lymphocytes<br>Trichinae: absent	N/A	N/A	-
Walker	1932	1 M	18	Delirium<br>Neck stiffness<br>Paraparesis<br>Bilateral mydriasis, absent light reflex<br>Absent biceps and knee reflexes	Normal	Brain edema, engorged capillaries<br>Normal meninges	N/A	Blood: leukocytosis with 97% polymorphonuclears (**without** eosinophilia)<br>Outcome: death
		1 F	18	Headache	-	N/A	N/A	Blood: leukocytosis with

				Neck stiffness<br>Positive Kerning sign<br>Pupils: slow light reaction<br>Diminished tendon reflexes				97% polymorphonuclears (without eosinophilia)<br>Eosinophilia occurred only in convalescence.<br>Outcome: improvement<br>Associated: bacterial infection
Filinski	1932	1		Right hemiplegia<br>Aphasia<br>Absence of deep and superficial reflexes.	-	Thrombosis of the superior sagittal sinus<br>Area of hemorrhagic softening in the left temporal lobe extending to the internal capsule<br>Larvae present in the brain	N/A	Outcome: death
Pund	1934	1 M	11	Drowsiness, followed by delirium and coma<br>The pupillary reflex was a little sluggish<br>Hypertonia of the lower limbs, especially of the flexor groups, that he was unable to stand or walk.<br>Diminished or absent reflexes<br>Absent Kernig sign	Pressure: 6 mmHg<br>Clear<br>35 cells/mm^3<br>Pandy negative	Diffuse congestion of the brain<br>Sections from the cortex, basal ganglions, medulla and cerebellum - minute inflammatory foci comprising glial cells and a few plasma cells, lymphocytes and polymorphonuclears, with an occasional endothelial leukocyte<br>Very rarely - syncytial mass with five or six nuclei, which resembled the nuclei of the glial cells. In the center of many of these foci a granular wormlike parasite was seen, the embryo of the trichina.<br>A few fragmented parasites<br>The foci were irregularly distributed in both the white and gray matter and sometimes were near a capillary. They were more numerous in the basal ganglions, medulla and cerebellum than in the cortex<br>The pia of the cortex and medulla - diffusely infiltrated with a few lymphocytes, plasma cells and polymorphonuclears, and a like infiltration occurred around and in the adventitia of the larger blood vessels of the cortex and of the basal ganglia. The infiltration of the vessels presented a tendency to be focal.<br>All of the blood vessels were engorged.<br>Many of the neurons in all sections - chromatolysis, pyknosis and	N/A	Associated: sickle-cell anemia.<br>Symptoms onset: 3 weeks after smallpox vaccination<br>Blood: leukocytosis **without** eosinophilia<br>Outcome: death

						sometimes neuronophagia.<br>Choroid plexus - normal		
Gordon	1935	1 F	14	Apathy<br>Drowsiness<br>Delirium<br>Optic fundi - slightly congested vessels without any alteration in the discs<br>Ptosis of the left eye<br>Kerning sign	Pressure: normal<br>Clear<br>30 cells/field, 95% lymphocytes, 5% polymorphonuclears.<br>Albumin: one-plus;<br>Globulin: reduced<br>Glucose: reduced.	Cerebral congestion and edema.<br>Toxic encephalosis.<br>The dura was tense and slightly congested.<br>The surface of the brain was slightly edematous;<br>The large veins were congested;<br>Distinct increase in the finer arterial subarachnoid ramification over the gyri.<br>The brain was of normal firmness.<br>Multiple sections of the cerebrum and cerebellum - a distinct hyperemia of the gray matter, which assumed a pink appearance throughout.<br>The white matter - punctate hyperemia of the severed small vessels.<br>The spinal fluid - slightly increased in amount<br>**Microscopic:**<br>Sections from parietal lobe - the pial membrane was slightly thickened with a sparse infiltration of mononuclears and an occasional macrophage.<br>Rarely - the cortical cells revealed slight nuclear chromatolysls.<br>The Virchow-Robin spaces - slightly widened.<br>The glia cells were slightly increased in number; this was most noticeable in the vicinity of the capillaries.<br>All cortical capillaries were immensely engorged and prominent throughout.<br>Some interstitial edema in all sections.	N/A	Absence of 3 generally accepted diagnostic criteria, gastrointestinal symptoms, muscular tenderness, and eosinophilia until the day preceding death.<br>Outcome: death – 19 days after the symptom's onset
Spink	1935	1F	42	Left facial palsy<br>Palatal reflex – absent<br>Right hemiplegia<br>Absent knee jerks<br>Babinski sign on the right	Normal	N/A	N/A	Blood: leukocytosis with eosinophilia<br>Outcome: improvement
Merritt	1936	1 F	17	Neck stiffness<br>Confusion, delirium<br>Headache	Pressure: increased<br>Clear, colorless<br>Cell free	N/A	N/A	Blood: leukocytosis with eosinophilia<br>Outcome:improvement

				Spastic left hemiplegia<br>Flaccid paralysis of the muscles of the left shoulder girdle, apparently of a lower motor neuron type<br>Biceps and triceps reflexes - absent on the left side and normally active on the right.<br>The radial periosteal reflex was diminished on the left.<br>The knee and ankle jerks - hyperactive on both sides, but more so on the left.<br>Sustained ankle clonus on the left and an unsustained ankle clonus on the right.<br>Bilateral Babinski sign<br>Absent abdominal reflexes<br>Slight weakness of the left lateral gaze, impaired convergence and upward and downward gaze	Protein: 17 mg<br>Glucose: 54 mg<br>Absent trichinae			
Blumer	1926	1 M	26	Neck stiffness<br>Confusion<br>Drowsiness<br>Delirium<br>Coma<br>Numbness of the left arm and leg<br>Constant, slow, side-to-side movement of both eyes. Pupils: equal in size, moderately wide, but reacted poorly to light<br>Left hemiplegia<br>Brisk tendon reflexes<br>Bilateral ankle clonus	Cells:0<br>Glucose: increased<br>Globulin: increased<br>Trichinae larvae: +	N/A	N/A	Blood: leukocytosis with eosinophilia<br>Outcome: improvement
Most	1937	1 F	42	Headache<br>Depression<br>Delirium<br>Unequal pupils, and reacting sluggishly to light and in accommodation<br>Left central facial palsy<br>Left upper limb paresis<br>Tendon reflexes brisker on the left<br>Left Babinski sign	Clear<br>Normal pressure<br>No globulin or cells<br>Absent Trichinella	N/A	N/A	Blood: leukocytosis with eosinophilia<br>Outcome: improvement
		1 M	48	Disorientation<br>Impaired memory, especially for more recent events, with a tendency to confabulate.	No globulin or cells<br>Absent Trichinella	**Macroscopic:** normal<br>**Microscopic:**<br>Granulomatous nodules that appeared as isolated and discrete	N/A	Blood: leukocytosis without eosinophilia<br>Outcome: death<br>Associated diseases:

Lack of inside
Acalculia
Mild anisocoria, but pupils reacted promptly to light and in accommodation
Absent biceps reflexes
Absent tendon reflex in the lower extremities
Tetraparesis

lesions scattered throughout the brain substance. They occurred most frequently in the subcortical white substance, especially in the areas subjacent to the cortex, quite commonly in the deeper layers of all parts of the cortex, only in scattered areas in the basal ganglia, midbrain and pons and rarely in the cerebellum, spinal cord and more superficial layers
Appearance of the parasite in the parenchyma of the brain
Evidence of pathologic alterations in and around the blood vessels
Perivascular infiltrations situated especially in the cortex, the adjacent subcortex and the basal ganglia. These changes were noted around the veins, as well as the smaller and larger arterioles. The perivascular infiltrations varied from a few accumulated cells in the Virchow-Robin spaces to dense accumulations many layers deep. The cells were almost always limited to these spaces and did not infiltrate the parenchyma.
No eosinophils were observed.
Only rarely was a parasite observed in the infiltrating elements around the vessels.
Generalized engorgement and hyperemia of blood vessels
Extreme engorgement of vessels just below the ependymal layer
Fragmented erythrocytes and pigment in the parenchyma in the vicinity of the vessels
Many vessels, especially in the white substance immediately below the cortex, had edematous spaces around them. The parenchyma adjacent to some of the larger vessels, especially in the midbrain, showed a limited area of vacuolation and had a racemose appearance.
The architectonics of the cortex was disturbed only in the regions

syphilis and chronic alcoholism

						where there were unusually diffuse infiltrations of cells<br>Numerous round and oval cavities were diffusely distributed in the subcortical<br>white substance and in the midbrain (etat crible)<br>No trichinas in the meninges<br>The veins and arteries of the pia-arachnoid were hyperemic and increased in number, especially in the infiltrated regions.		
Evers	1939	1 M	25	Drowsiness, followed by coma<br>Dilated pupils with sluggish reaction to light.<br>Transient divergent strabismus.<br>Optic fundi:<br>- right eye – edema of the disk and the<br>surrounding retina, with exudate, slight perivascular<br>change along the superior temporal vein.<br>- left eye mild edema of the disk and of the surrounding retina<br>Increased muscular tone in upper limbs<br>Bilateral ankle clonus<br>Bilateral Babinski sign	Pressure: normal<br>Clear<br>10 lymphocytes / mm^3<br>Proteins: absent<br>Glucose: normal<br>Motile larvae +	N/A	N/A	Blood: leukocytosis with eosinophilia<br>Outcome: improvement
Huston	1941	1 M	19	Drowsiness<br>Somnolence<br>Left arm spastic<br>Brisk knee and ankle jerks<br>Bilateral ankle-clonus<br>Bilateral Babinski sign.	Normal	N/A	N/A	Blood: leukocytosis with eosinophilia<br>Outcome: improvement
		1 F	69	Drowsiness<br>Delirium<br>Frequent crying<br>Loss of tone in facial muscles<br>Flaccid arms with no voluntary movement or power<br>Biceps jerks present on both sides; supinator and triceps jerk absent<br>Both legs flaccid, particularly right<br>Knee and ankle jerks present<br>Bilateral Babinski sign	Normal	Sections of frontal, parietal, temporal and occipital cortex, centrum semi-ovale, basal ganglia, pons, medulla and cerebellum show neither larvae nor inflammatory foci.	N/A	Blood: leukocytosis with eosinophilia<br>Outcome: death
Sheldon	1941	1 M		The patient lied curled up in	Normal or reduced	N/A		Outcome: improvement

				bed, with retracted head,<br>photophobia and a piercing<br>meningeal cry<br>Impaired consciousness<br>Irritability<br>Positive Kernig sign<br>Bilateral Babinski sign	pressure<br>Normal findings			
		1		Encephalitis clinical signs				
		1		Depression				
		1 F		Cerebellar symptoms<br>Typical decubitus of encephalitis<br>lethargica, but without the ocular<br>palsies<br>Lethargy – for a time the patient<br>is indifferent to the surroundings,<br>and in particular resent mental<br>effort, such as answering<br>questions.<br>Sleepiness				
		3		Focal brain damage – unilateral<br>cerebellar signs				
Wyrens	1941	1		Right hemiparesis	-	N/A	N/A	Eosinophilia<br>Outcome: improvement
Campbell	1947	1 M	30	Left hemiparesis<br>Ataxia<br>Psychosis<br>Delirium	Normal	N/A	N/A	Outcome: improvement
Battle	1948	1 F	3	Right hemiplegia	Normal	N/A	N/A	Outcome: improvement
MacAndrew	1948	1 F	39	Slowed mentation<br>Depression<br>Right central facial palsy<br>Right footdrop<br>Sensory loss with L4-L5-S1<br>Diminished right ankle and knee<br>jerks<br>Absent Babinski sign	Normal	N/A	N/A	Blood: leukocytosis with<br>eosinophilia<br>Subungual " splinter"<br>hemorrhages<br>Outcome: improvement
Skinner	1948	1 F	60	Neck stiffness<br>Apathy<br>Confusion<br>Drowsiness<br>Left flaccid hemiparesis<br>Right Babinski sign	Pressure: 110 mm H_2O<br>Xanthochromia<br>Cells: red blood cells –<br>700/mm^3<br>Protein: 11mg<br>Glucose normal	N/A	N/A	Blood: leukocytosis with<br>eosinophilia<br>Outcome: improvement
		1 M	40	Agitation<br>Anxiety<br>Disorientation<br>Delirium<br>Left hemiparesis<br>Left Babinski sign<br>Tendon reflexes: brisk knee and<br>ankle jerks	Normal	N/A	N/A	Blood: leukocytosis with<br>eosinophilia<br>Outcome: improvement

				Impaired position sense in the left body				
Bruno	1950	1 M	16	Neck stiffness<br>Delirium	Normal	N/A	N/A	Outcome: improvement
McCabe	1951	1 F	19	Disorientation<br>Neck stiffness<br>Pupils: dilated but equal and reacted sluggishly<br>to light and in accommodation	Pressure: 110 mm H2O<br>Clear<br>Trichinella absent<br>Cells: 0<br>Protein: normal<br>Glucose: normal	N/A	N/A	Blood: leukocytosis **without** eosinophilia<br>Outcome: death
		1 M	40	Auditory and visual hallucinations with paranoid grandiose and persersecutory delusions<br>Oriented in all spheres,<br>Emotional blunting and dissociation	Normal	N/A	N/A	Blood: leukocytosis with eosinophilia<br>Outcome: improvement
Davis	1951			Agitation<br>Confusion<br>Disorientation	Normal<br>Trichinella larvae absent	N/A	N/A	Blood: leukocytosis with eosinophilia<br>Outcome: improvement
Helfand	1952	1 F	33	Headache<br>Paraparesis<br>Visual loss	103 white blood cells	N/A	N/A	Outcome: improvement
Brimblecombe	1952	1 M	5	Seizures: petit mal, grand mal	Normal	-	N/A	No eosinophilia
Scott	1952	1 M	28	Neck stiffness<br>Disorientation<br>Confusion	Clear<br>Pressure: 225mm H2O<br>Protein: normal<br>2 red cells	N/A	N/A	Blood: leukocytosis with eosinophilia<br>Outcome: improvement
		1 M	26	Headache<br>Confusion<br>Stupor<br>Brisk tendon reflexes<br>Bilateral ankle clonus<br>Bilateral Babinski sign	Clear<br>Protein: normal<br>3 lymphocytes	N/A	N/A	Blood: leukocytosis with eosinophilia<br>EEG: paroxysmal 5-6/sec wave bilaterally<br>Outcome: improvement
		1 M	33	Headache<br>Episode of consciousness loss<br>Somnolence<br>Meningeal signs<br>Eyes deviated to the right<br>Left central facial palsy<br>Tetraparesis (left > right)<br>Spasticity (left > right)<br>Brisk tendon reflexes (left > right)<br>Bilateral Babinski sign	Clear<br>Pressure: 180 mm H2O<br>Protein: normal<br>No cells	N/A	N/A	Blood: leukocytosis with eosinophilia<br>Outcome: improvement
Hurd	1953	1 M	40	Neck stiffness<br>Confusion<br>Impaired recent memory<br>Right hemiparesis<br>Diminished knee and ankle jerks	Normal pressure<br>Normal proteins and glucose<br>4 lymphocytes	Head Rx: without calcifications	N/A	Blood: leukocytosis without initial eosinophilia.<br>Eosinophilia appeared after leucocytes

				Right Babinski sign				decreased<br>Outcome: improvement
Roehm	1953	1 M	34	Amnesia, mainly for recent memories (past 3 weeks), but no confabulation<br>Impaired ability to learn new things<br>Disorientation<br>Tetraparesis<br>Radial neuritis<br>Insomnia with agitation	Pressure 180 mm<br>Protein, 52 mg/dl<br>Negative mastic<br>Cells: normal	N/A	N/A	Blood: leukocytosis without initial eosinophilia<br>EEG: mild irregularities, no cortical dysfunction<br>Outcome: improvement
Semple	1954	1		Cerebellar signs<br>Monoplegia	Normal	N/A	N/A	Outcome: improvement
		1		Coma<br>Meningeal position and cry	Normal	N/A	N/A	Outcome: improvement
		1		Semi-comatose	Normal	N/A	N/A	Outcome: improvement
		1		Semi-comatose<br>Monoplegia	Normal	N/A	N/A	Outcome: improvement
		9		Minor psychic disturbances, with either delusions or an obvious personality change.<br>The personality changes mainly took the form of the patient behaving in an unusual manner. One sang constantly; another climbed out of bed and calmly urinated on the floor.<br>Hallucinations and visual disturbances were not reported.	-	N/A	N/A	Outcome: improvement
Freedman	1954	1 F	47	Headache<br>Personality changes<br>Depression<br>Apathy<br>Disorientation<br>Neck stiffness<br>Diminished knee and ankle jerks<br>Right arm weakness<br>Cerebellar signs	Pressure: 125 mm.<br>Negative white cells<br>Red cells: many<br>Proteins: 620 mg%<br>Glucose: 91 mg% Larvae of Trichinella spiralis - not searched for.	N/A	N/A	Blood: leukocytosis with eosinophilia<br>Associated: bacterial urinary infection<br>Outcome: improvement
Marcus	1955	1 M	30	Dysphagia (as seen in bulbar or pseudobulbar disease)<br>Tetraparesis<br>Diminished tendon reflexes<br>Right Babinski sign	Normal pressure<br>Clear<br>Glucose: 70 mg/dl<br>Protein: 18 mg/dl	N/A	N/A	Blood: leukocytosis **without** eosinophilia; only on one occasion eosinophilia was present (12%)<br>Associated: bacterial urinary infection<br>EMG: severe and widespread lower motor neuron changes<br>Outcome: improvement
Kershaw	1956	1 F		Dizziness	-	Absent Larvae in the brain	N/A	

				Insomnia				
		1 F		Cerebellar symptoms<br>Hemiplegia	-			
		1 F		Dizziness<br>Lethargy<br>Anxiety<br>Weakness of hands	-			
Leitner	1956	1 M	51	Stupor<br>Somnolence	Normal	N/A	N/A	Outcome: improvement
		1 M	42	Neck stiffness<br>Somnolence	-	N/A	N/A	Outcome: improvement
Snape	1956	1 F	22	Psychosis<br>Hallucinations	Normal	N/A	N/A	Outcome: improvement
Guattery	1956	1 F	18	Delirium	-	Encephalitis	N/A	Blood: leukocytosis<br>Outcome: death
Meltzer	1957	1 M	43	Headache<br>Confusion<br>Disorientation<br>Lethargy<br>Pupils – equal, sloe light reaction<br>Motor aphasia<br>Left wrist drop<br>Tendon reflexes left > right<br>Bilateral Babinski sign	Pressure: 110 mm<br>Clear<br>Cells: 0<br>Glucose: normal<br>Protein: 19 mg/100 cc	N/A	Skull X-Ray: normal	Blood: leukocytosis with eosinophilia<br>Outcome: recovery
Terplan	1957	1 B	8	Lethargy	Cells: 755<br>Protein: 466 mg.	**Macroscopic:** uniform swelling of both cerebral hemispheres and of the cerebellum, with compression of the fourth ventricle and protrusion of the tonsils into the foramen magnum.<br>**Microscopic:** dense inflammatory reaction in the cerebellum, predominantly eosinophilic; an occasional free trichinella larva was found in the thalamus.	N/A	Outcome: death
Chase	1957	1 M	27	Headache<br>Psychomotor epileptic seizures<br>Dysphagia<br>Tetraparesis<br>Diminished tendon reflexes with absent knee reflexes	Clear<br>Protein: 28 mg/dl<br>Glucose: 100 mg/dl	-	N/A	Blood: leukocytosis with eosinophilia<br>Outcome: death
Mavor	1958	1 F	23	Headache<br>Lethargy<br>Impaired serial subtractions<br>Optic fundi: engorgement of veins, blurring of the disc margins with hyperemia and radial striations; bilateral swelling of the optic disc<br>Flaccid paresis of left upper limb	Pressure: 140 mm H2O<br>Clear<br>Cells: 1 red, 4 white /mm^3<br>Protein: 31 mg/dl	N/A	Skull X-ray: normal	Blood: leukocytosis with eosinophilia<br>EEG: medium-voltage, arrhythmic activity in all areas, 4-15/sec, intermittent high-voltage 2/sec rhythmic discharges in the left frontal pole, with spread

				Bilateral brisk tendon reflexes<br>(left > right)<br>Bilateral ankle clonus<br>Absent Babinski sign				to the midline and right<br>areas<br>Outcome: improvement
Levine	1958	1 F	26	Headache<br>Delirium<br>Right hemiparesis	Normal	N/A	N/A	Outcome: improvement
Greenstein	1958	1 M	6	Neck stiffness<br>Generalized clonic convulsions<br>Stupor<br>Kerning sign positive<br>Bilateral Babinski sign	Pressure: 260 mm H2O<br>Clear<br>Cells: lymphocytes; Pandy<br>test negative<br>Protein: 24 mg/100 ml<br>Negative cultures	N/A	N/A	Blood: leukocytosis with<br>mild eosinophilia<br>Associated diseases:<br>febrile convulsions,<br>retardation of<br>development<br>Outcome: improvement
Dalessio	1961	1 F	45	Headache<br>Blurring of vision<br>Right upper limb paresis with<br>paresthesia followed by<br>tetraparesis<br>Lethargy<br>Coma<br>Generalized hyporeflexia<br>Bilateral Babinski sign<br>Irritability<br>Insomnia<br>Amnesia for recent events	Pressure: normal<br>Protein: normal<br>Glucose: normal<br>Red blood cells: 50	N/A	N/A	Blood: leukocytosis with<br>eosinophilia<br>Splinter hemorrhages<br>in her fingernail beds.<br>EEG: diffuse<br>abnormalities<br>Outcome: improvement
Grey	1962	1 M	28	Headache<br>Neck stiffness<br>Disorientation<br>Lethargy<br>Apathy<br>Uncontrolled bouts of crying<br>Photophobia<br>Transitory diplopia<br>Ptosis of the left eyelid<br>Scintillating scotomata<br>Positive Romberg sign<br>Ataxia (particularly in the right<br>arm and left lower limb)<br>Paresthesia and weakness of left<br>lower limb<br>Left hemiparesis<br>Spasticity of left lower limb<br>Jerky, rhythmic movements of<br>the left foot<br>Involuntary movements of the<br>upper extremities<br>(hemiballismus)<br>Brisk lower limbs tendon reflexes<br>Bilateral ankle clonus	Pressure: 120 mm H2O<br>Clear<br>Cells: 4 white blood cells<br>Protein 27 mg/100 ml,<br>Glucose: 70 mg/100 ml<br>Absent Trichinella	N/A	Skull roentgenograms:<br>normal	Blood: leukocytosis with<br>eosinophilia<br>Splinter hemorrhages<br>beneath nails<br>Outcome: improvement

				Bilateral Babinski sign				
		1 M	42	Neck stiffness<br>Change in personality<br>Lethargy<br>Emotional lability<br>Insomnia<br>Apprehension.<br>Short period of blurred vision and scintillating scotomata<br>Nystagmus on left lateral gaze<br>Positive Romberg sign Dysmetria in left upper limb<br>Left hemiparesis<br>Brisk tendon reflexes in lower limbs<br>Left Babinski sign	Pressure 160 mm<br>Clear<br>Cells: 0<br>Protein: 19 mg/100 ml.	N/A	N/A	Blood: leukocytosis with eosinophilia<br>Splinter hemorrhages<br>Associated disease: Entamoeba histolytica (demonstrable in random stool specimens)<br>Outcome: improvement
		1 M	34	Headache<br>Lethargy<br>Disorientation<br>Weakness of the left arm<br>Brisk tendon reflexes on the left side<br>Left ankle clonus<br>Left Babinski sign	Pressure 140 mm<br>Clear<br>Cells: 6 mononuclear cells/mm3<br>Protein: 30 mg/100 ml.	N/A	N/A	Blood: leukocytosis with eosinophilia<br>Outcome: improvement
Perot	1963	1 F	42	Drowsiness<br>Memory impairment<br>Bizarre mental attitude<br>The present illness precipitated a mental breakdown in a predisposed patient.	N/A	N/A	N/A	Blood: leukocytosis with eosinophilia<br>Associated diseases: schizophrenia<br>EEG: diffuse and moderately severe disturbance involving cortical and subcortical structures<br>Outcome: improvement
		1 F	36	Headache<br>Drowsiness<br>Confusion<br>Delirium<br>Left hemiparesis<br>Bilateral equivocal Babinski sign<br>Right hemianopsia<br>Motor aphasia<br>Apraxia	N/A	N/A	N/A	Blood: leukocytosis with eosinophilia<br>EEG: marked cerebral disturbance involving both cortical and subcortical structures with some evidence for a projected abnormality involving the left side more than the right.<br>Associated: acute pelvic inflammatory disease<br>Outcome: improvement
		1 M	13	Episode of memory loss, lasting several hours<br>Impaired memory for recent	Pressure: 155 mm H2O<br>Clear<br>2 lymphocytes	N/A	Skull Roentgenology normal	Blood: leukocytosis with eosinophilia<br>EEG: mild bilateral

				events<br>"Staring spells"<br>The patient said that things happened as if "in a dream."<br>Apathy<br>Depression<br>Bizarre behavior<br>Somnolence<br>Episodes of bizarre behavior after initial illness	Protein 12 mg/dl.<br>Glucose 63 mg/dl<br>Absent Trichinella			abnormal activity mainly from the parieto-occipital and temporal regions<br>Outcome: improvement
Barr	1966	1 F	32	Lethargy<br>Confusion<br>Dizziness	Normal	N/A	N/A	Blood: leukocytosis with eosinophilia<br>EEG: diffuse anterior brain-stem disturbance.<br>Subungual splinter hemorrhages<br>Outcome: improvement
		1 F	46	Headache<br>Severe paranoid reaction<br>Optic fundi: retinal vein congestion.<br>Bilateral sixth nerve palsy	Normal	N/A	N/A	Blood: leukocytosis with eosinophilia<br>Outcome: improvement
Kennedy	1966	1 F	39	Headache<br>Confusion<br>Irritability<br>Left central facial palsy<br>Left hemiplegia<br>Generalized hypertonia<br>Left Babinski sign	Normal	N/A	N/A	Blood: leukocytosis with eosinophilia<br>Outcome: improvement
Corridan	1969	6		Memory loss	-	N/A	N/A	-
		1		Facial paralysis	-	N/A	N/A	-
		1		Hemiplegia	-	N/A	N/A	-
		1		Delirium	-	N/A	N/A	-
Kramer	1972	1 F	17	Impaired abstract thinking<br>Acalculia<br>Episodes of anosognosia<br>Dizziness<br>Left lateral rectus palsy (sixth cranial nerve)<br>Left central facial palsy<br>Left hemiparesis<br>Pain and paresthesia over the left side of the body<br>Brisk tendon reflexes (left > right)<br>Left sustained ankle clonus<br>Babinski sign – left plantar indifference	Normal	N/A	Skull X-Ray: normal	Blood: leukocytosis with eosinophilia<br>EEG: diffusely abnormal<br>Splinter hemorrhages under the nails<br>Outcome: improvement
Davis	1976	1 M	26	Optic fundi: Numerous punctate and splinter retinal hemorrhages in both eyes	Slightly traumatic<br>Red blood cells: 10,300<br>White blood cells: 24 (21	N/A	N/A	Blood: leukocytosis with eosinophilia<br>Outcome: improvement

				Bilateral sixth cranial nerve palsy<br>Absent tendon reflexes<br>Muscular hypotonia	polymorphonuclear leukocytes, 3 lymphocytes)<br>Protein: 11.3 mg/dl<br>Glucose 52 mg/dl			
Gross	1979	1 F	35	Mental slowness<br>Depression<br>Change in perception of auditory and visual images and some impairment<br>of thought processes<br>Confusion<br>Optic fundi: multiple bilateral flame-shaped retinal hemorrhages, without vitreous extension<br>Mild bilateral ptosis<br>Diminished tendon reflexes	-	N/A	N/A	Blood: absence of significant eosinophilia until three to four weeks into the illness<br>Outcome: improvement
Evans	1982	1 M	46	Headache<br>Optic fundi: papilledema<br>Generalized tonic-clonic seizures	Pressure of 300 mm H2O	N/A	Brain CT scan: hypodense area<br>with some contrast enhancement compatible with an acute<br>infarction in the area of the left sylvian fissure<br>Angiogram: thrombosis<br>of the superior sagittal sinus	Blood: leukocytosis with eosinophilia<br>Outcome: improvement
Froscher	1982	1 M		Psycho-motor epileptic seizures	-	-	-	-
Gay	1982	1 F	50	Headache<br>Coma<br>Photophobia<br>Seizures<br>Flaccid left upper limb paresis<br>Signs of brainstem compression	Trichinella larvae: -	**Macroscopic:** Cerebral edema, causing uncal and cerebellar herniation and necrosis.<br>The cortical venous system and dural sinuses were extensively thrombosed.<br>In the brain substance were areas of hemorrhage and infarction in a pattern resulting from venous occlusions<br>**Microscopic:**<br>No signs of vasculitis<br>Mild meningitis of lymphocytes and eosinophils, including involvement of Virchow-Robin spaces.<br>In cortical areas not included in the regions of infarction and hemorrhage were small foci of necrosis, infrequently in the form of linear clefts<br>Trichinella larvae: absent	Serial brain CT: enlarging areas of right parietal hemorrhage.	Blood: leukocytosis with eosinophilia<br>Outcome: death
Lyon-Caen	1982	1		Behavioural disorders	-	N/A	Brain CT: transient low-	Outcome: improvement

				Tetraparesis<br>Oculomotor paralysis.			density areas in the white matter.	
Ancelle	1985	4		Oculomotor impairments<br>Dizziness<br>Impairment of visual field<br>Dysesthesia<br>Paresis	-	-	-	Outcome: 2 improvement, 2 death
Ellrodt	1987	1 M	58	Quadriparesis<br>Brisk tendon reflexes<br>Bilateral Babinski sign	Normal pressure<br>Clear<br>Cells: 0<br>Protein: normal<br>Glucose: normal<br>Antibodies to Trichinella +	N/A	CT: multifocal low-density lesions in the corona radiata. Nodular enhancement of these lesions and gyral enhancement	Blood: leukocytosis with eosinophilia<br>EEG: bilateral, predominantly left, delta, and theta waves in the frontal area.<br>Outcome: improvement
		1 M	56	Confusion<br>Euphoria<br>Restlessness<br>Gait disturbance<br>Upper limb weakness<br>Impaired sensation	Normal	N/A	CT: nonenhanced low-density areas in the left corona radiata; low-density areas in the anterior part of the right hemisphere.<br>**MRI:** bilateral high signal-intensity lesions of the white matter and in the periventricular area	Blood: eosinophilia<br>EEG: frontal and temporal theta wave<br>Outcome: improvement
		1 M	23	Confusion<br>Transient dysesthesia in the left lower limb<br>Right upper limb monoplegia<br>Brisk right-sided tendon reflexes	Normal	N/A	Contrast-enhanced CT scan: normal	Blood: eosinophilia<br>EEG: bilateral slow waves, predominantly right, affecting the temporal and occipital regions.<br>Outcome: improvement
Ryczak	1987	1 F	62	Confusion<br>Disorientation<br>Cortical blindness<br>Quadriplegia	Normal	N/A	CT scan: normal<br>Angiogram: normal	Blood: leukocytosis with initial eosinophilia.<br>Normal eosinophiles after 3 weeks, despite neurological aggravation<br>Outcome: improvement
Vega	1987	1 M	9	Headache<br>Neck stiffness<br>Mental slowness<br>Dysarthria<br>Ataxia	Normal	N/A	N/A	Blood: leukocytosis with eosinophilia<br>Outcome: improvement
		1 M	12	Neck stiffness<br>Bradylalia<br>Obnubilation<br>Confusion<br>Hypertonia of lower limbs<br>Brisk lower limb tendon reflexes	Normal	N/A	N/A	Blood: leukocytosis with eosinophilia<br>Outcome: improvement
Kreel	1988	1 F	8	Left sided focal seizures<br>Left Babinski sign	-	N/A	Skull radiograph: enlargement of the right middle fossa with elevation	Blood: leukocytosis with eosinophilia<br>Associated diseases:

							of the lesser wing of the sphenoid and slight enlargement of the superior orbital fissure<br>CT: two hypodense areas in the white matter, one in the right postero-superior region of parietal lobe and the other adjacent to the falx in the right frontal lobe<br>A small enhanced ring lesion of 8 mm was found adjacent to each of the hypodense edematous areas as well as a similar lesion in the left posterior parietal region adjacent to the falx	neonatal meningitis, with complete remission<br>Outcome: improvement
		1 M	43	Headache	-	N/A	CT: 3 small calcified rings of 3-8 mm, one adjacent to the anterior aspect of the right frontal horn and the other two near the left posterior horn of the lateral ventricles. There was slight contrast enhancement of the anterior lesion, but no surrounding edema.	Blood: eosinophilia<br>Outcome: improvement
Lopez-Lozano	1988	1 M	50	Neck stiffness<br>Bilateral peripheral facial palsy<br>Tetraparesis<br>Generalized brisk tendon reflexes<br>Positive Kerning sign	Normal pressure<br>Clear<br>Lymphocytes:150 cells/ mm3<br>Proteins: 160 mg/dl<br>Glucose: 52 mg/dl	N/A	N/A	Blood: leukocytosis with eosinophilia<br>Outcome: improvement
Fourestie	1993	1 M	54	Headache<br>Mutism<br>Decreased cognitive abilities<br>Impaired memory<br>Temporal and spatial disorientation<br>Nocturnal agitation<br>Gait disturbance (which could not be accounted for by demonstrable sensory or motor deficits)<br>Right hemiparesis<br>Left Babinski sign	Cells: 2/mm3<br>Protein: 48 mg/dl<br>Glucose: normal	N/A	**CT**: multiple very small hypodense areas in the centrum semi-ovale of both cerebral hemispheres<br>Absent enhancing lesions<br>**MRI:** (T2 weighted sequence) high signals in the periventricular white matter.	Blood: eosinophilia<br>EEG: fluctuating bifrontal slowing<br>of the electrocortical activity<br>Outcome: improvement
		1 M	55	Headache<br>Confusion<br>Spatial and temporal	Cells: 0<br>Protein: 76 mg/dl<br>Glucose: normal		**CT:** focal hypodense area in the right frontal lobe cortex and multiple	Blood: eosinophilia<br>EEG diffuse slowing of cortical activity

				disorientation<br>Intellectual impairment<br>Behavioural abnormalities<br>Left Homer's sign<br>Gait problems<br>Bilateral ataxia<br>Right Babinski sign			bilateral small hypodense areas in the hemispheric white matter.	Outcome: improvement
		1 M	72	Headache<br>Confusion<br>Aphasia<br>Left sixth nerve palsy<br>Right hemiparesis<br>Right Babinski sign	Cells: 1 cell/mm3<br>Protein: normal<br>Glucose normal	Multiple small ischemic cavities in the hemispheric white matter and pons<br>Small cortical infarcts. Both lesions were < 1 cm and were pale or slightly hemorrhagic. Neither inflammatory infiltrates nor remnants of Trichinella<br>larvae or microglial nodules were found.<br>Arteriolar microthrombi were found in, or close to, most ischemic areas. Some vessels showed non-inflammatory necrosis.	**CT:** unenhanced hypodensities in the white matter	Blood: eosinophilia<br>Associated conditions: tremor<br>Outcome: death
		6		Spatial-temporal disorientation 4/6<br>Intellectual deficit 2/6<br>Memory disturbances 3/6<br>Frontal syndrome 3/6<br>Behavioral disturbances 0/6<br>Somnolence 1/6<br>Tetraparesis 2/6<br>Headache 3/6<br>Oculomotor dysfunction 1/6<br>Central visual deficit: 3/6<br>Aphasia 1/6<br>Cerebellar syndrome 1/6<br>Central sensory deficit: 1/6<br>Myoclonus 1/6	Normal 3/6<br>Protein: increased 2/6	N/A	CT:<br>Large cerebral infarction 0/6<br>Small hypodensities 6/6<br>Enhancement: 1/6	Blood: eosinophilia<br>Outcome: improvement
Louthrenoo	1993	1 M	34	Headache<br>Diplopia<br>Bilateral lateral rectus muscle paresis<br>Gaze-evoked nystagmus<br>Dysphagia<br>Tetraparesis<br>Bilateral pain and touch hypostasis<br>Bilateral decreased tendon reflexes	Clear<br>Mononuclear cells: 20 cells/mm3<br>Glucose: 67 mg/dl<br>Protein: 60 mg/dl	N/A	N/A	Blood: absent leukocytosis, **no** eosinophilia<br>Associated diseases: HIV, mononeuropathy multiplex<br>Outcome: improvement
El Koussa	1994	1 M	43	-	-	-	**MRI and angio-MRI**: right Rolandic hemorrhagic infarct and thrombosis of	Outcome: death

							the superior sagittal and left lateral sinuses	
Clausen	1996	1 F	56	Neck stiffness<br>Coma	Initially normal<br>When patient was comatose:<br>Protein: 0.90-1.13 g/l	N/A	**CT:** leukoencephalopathy, and small hypodense areas	Blood: leukocytosis with eosinophilia<br>Eosinophilia was absent at admission<br>Outcome: improvement
Feydy	1996	1 M	60	Headache<br>Right arm weakness<br>Aphasia	Cells:0<br>Protein: 0.71 g/l	N/A	**CT:** one small low-density area in the left centrum semiovale, and one in the right centrum semiovale, both without enhancement<br>**MRI:** the bilateral centrum semiovale lesions with high signal on proton density- and T2-weighted images; they gave slightly low signal on T1-weighted images and did not enhance with Gd-DTPA. A high signal area was evident in the left precentral gyrus on proton density- and T2 images. There were subtle areas with the same signal characteristics in the right precentral and middle frontal gyri. There was focal gyriform enhancement of the left cerebral lesion and focal nodular enhancement in the right frontal cortical lesions.	Blood: eosinophilia<br>Outcome: improvement
De Graef	2000	1		-	-	-	**MRI:** multifocal hyperintensities on T2 images	-
Knejevic	2001	1	30	Somnolence<br>Confusion<br>Disorientation<br>Right hemiparesis	Normal	N/A	**MRI:** multifocal unspecific changes	EEG: moderate cerebral dysfunction<br>Outcome: improvement
Gelal	2005	1 F	61	Neck stiffness<br>Headache<br>Somnolence<br>Confusion<br>Behavioral changes<br>Apathy<br>Right hemiparesis.<br>Ataxia,	-	N/A	**MRI:** T2 and FLAIR sequences revealed several cortical and subcortical millimetric nodular lesions with slight signal increase<br>Images in T1 sequences were normal with no	Blood: leukocytosis with eosinophilia<br>Outcome: improvement

				Hyperesthesia dominant in the right hand			contrast enhancement<br>Isotropic singleshot echo planar DW images revealed numerous lesions located in the subcortical and periventricular white matter of cerebral hemispheres, frontoparietal cortex, and splenium of corpus callosum. The lesions representing restricted diffusion were less than 1 cm in diameter. They were distributed mainly in the border-zones or distal fields of major cerebral arteries.<br>Sequential evaluation of conventional and diffusion MRI data indicates that multifocal lesions were related to multiple infarctions rather than true inflammatory infiltration of the brain parenchyma.	
Langner	2007	1 M	43	Headache<br>Euphoria<br>Cortical blindness<br>Bilateral ptosis<br>Anisocoria<br>Tetraparesis	Cells: 0	Stereotactically guided **brain biopsy** of a right frontal lesion showed active vasculitic changes with focal fibrinoid necrosis of the vessel wall and deposits of eosinophils.	**CT:** normal<br>**MRI:** DWI sequences revealed multiple hyperintense lesions at the border zone of the middle cerebral artery and in both cerebellar hemispheres and the inferior vermis; all lesions were hypointense on apparent diffusion coefficient (ADC) maps.<br>On T2-weighted and FLAIR images the lesions presented high signal intensity.<br>Intra-arterial angiography of the intracranial arteries was without evidence of vasculitis changes<br>The follow-up MRI (after 15 days) showed a decrease in the number of lesions on DWI, with peripheral enhancement after	Blood: leukocytosis with eosinophilia<br>Outcome: improvement

							intravenous Gd-DTPA indicating blood–brain-barrier breakdown.	
Madariaga	2007	1 F	53	Disorientation<br>Apathy<br>Aphasia<br>Coma<br>Left upper limb paresis	Red blood cells: multiple (traumatic puncture)<br>Neutrophiles: 8<br>Protein: 64 mg/dL<br>Glucose: 140 mg/dL	N/A	**CT:** normal<br>**MRI:** nonspecific white matter changes	Blood: Leukocytosis **without** eosinophilia<br>Eosinophilia appeared 3 days later<br>Outcome: improvement
Neghina	2011	1 F	65	Muscle fasciculations<br>Left hemiplegia	-	-	N/A	Blood: eosinophilia
		1 M	26	Left hemiparesis	-	N/A	**N/A**	Blood: eosinophilia without leukocytosis
		1 M	33	Right hemiparesis	-	N/A	**N/A**	Blood: absent leukocytosis, **without** eosinophilia
		1 M	28	Encephalitis<br>Right arm monoplegia	-	N/A	**N/A**	Blood: leukocytosis with eosinophilia
		1 F	54	Encephalitis	-	N/A	**N/A**	Blood: leukocytosis with eosinophilia
		1 F	24	Encephalitis	-	N/A	**N/A**	Blood: leukocytosis
		1 F	17	Meningeal signs	-	N/A	**N/A**	Blood: leukocytosis with eosinophilia
McDonald	2014	1 M	41	Headache<br>Confusion<br>Somnolence<br>Impaired concentration<br>Aphasia<br>“Red spots” briefly appearing in the visual field<br>Left face numbness<br>Left arm numbness and weakness<br>Ataxia<br>Normal tendon reflexes	Erythrocytes: 9.5/mm3<br>Leukocytes 3.6/mm3<br>Protein: 34 mg/dL<br>Glucose: 57.6 mg/dL<br>Negative Gram stain and culture.	N/A	**CT:** areas of decreased attenuation bilaterally in the periventricular regions of the frontal lobes.<br>**MRI:** multiple bilateral areas of increased signal intensity in the deep white matter and gray–white matter interface of the frontal and parietal lobes bilaterally, with very few lesions seen in the occipital and temporal lobes and the cerebellum on FLAIR and T2-weighted scans. DWI scans demonstrated restriction in some of the deep white matter lesions, indicating ischemia.<br>Lesions were slightly hypointense on unenhanced T1 images and there was no pathologic blooming artifact on T2 gradient echo sequences that would have suggested blood	Blood: leukocytosis with eosinophilia<br>Outcome: improvement

							products.<br>Following contrast administration, there was an intense rim of enhancement in most lesions.	
Moscatelli	2014	1 F	14	Weakness of the soft palate<br>Left facial hemiparesis<br>Tetraparesis,<br>Lower limb clonus	Normal	N/A	**CT:** hypodense, multifocal, small lesions in the cortex and white substance<br>**MRI:** multifocal lesions hypointense in T1 sequences and hyperintense in T2-weighted images<br>The lesions were without mass effect, situated in the white substance and in the periependymal region, and enhanced contrast.	Blood: leukocytosis with eosinophilia<br>Outcome: improvement
Batzlaff	2014	1 F	72	Confusion<br>Short term memory impairment<br>Visual hallucinations<br>Anisocoria	-	N/A	**N/A**	Blood: leukocytosis with eosinophilia<br>Associated diseases: IgG deficiency requiring monthly intravenous immunoglobulin infusions<br>Outcome: improvement
Dalcin	2017	1 M	41	Headache<br>Optic fundi: bilateral papilledema<br>Blurred vision<br>Bilateral sixth cranial nerve palsy	N/A	N/A	**CT**: acute cerebral sinus venous thrombosis<br>**MRI:** a partially occlusive thrombus in the superior sagittal sinus and complete occlusion into the left transverse sinus.<br>The inferior sagittal sinus and straight sinuses were also found to have thrombi within them	Blood: normal leukocytes, **without** eosinophilia<br>Outcome: improvement
Rosca	2018	1 M	59	Drowsiness<br>Cognitive impairment Behavioral disturbances<br>Receptive aphasia<br>Tetraparesis<br>Globally diminished deep tendon reflexes	N/A	N/A	**CT:** normal<br>**MRI:** multiple nodular lesions, with high signal in T2 and FLAIR sequences, located relatively symmetrically, in the deep white matter and grey-white matter interface of cerebral hemispheres and the cerebellum (border zone lesions).<br>No contrast enhancement	Blood: leukocytosis with eosinophilia<br>Outcome: improvement
Mitrovic	2019	1 F	51	Confusion	Normal	N/A	**CT:** hypodense lesions	Blood: leukocytosis with

	Apathy<br>Right central facial paresis Right hemiparesis<br>Brisk brachioradialis, triceps and patellar reflexes<br>Babinski sign: indifference on the right side	Trichinella larvae: absent		bilaterally in the parietal region, with no signs of compression.<br>**MRI:** bilateral white matter lesions in the cerebral hemispheres (watershed zones ACA and ACM), lesions in the right thalamus and infratentorially (including cerebellum) on the left side.	eosinophilia<br>EEG: normal<br>Outcome: improvement
